# Clinical, imaging, biochemical and molecular features in Leigh syndrome: a study from the Italian network of mitochondrial diseases

**DOI:** 10.1186/s13023-021-02029-3

**Published:** 2021-10-09

**Authors:** Anna Ardissone, Claudio Bruno, Daria Diodato, Alice Donati, Daniele Ghezzi, Eleonora Lamantea, Costanza Lamperti, Michelangelo Mancuso, Diego Martinelli, Guido Primiano, Elena Procopio, Anna Rubegni, Filippo Santorelli, Maria Cristina Schiaffino, Serenella Servidei, Flavia Tubili, Enrico Bertini, Isabella Moroni

**Affiliations:** 1 Department of Pediatric Neuroscience, Fondazione IRCCS Istituto Neurologico “Carlo Besta”, Milan, Italy; 2 Center of Translational and Experimental Myology, IRCCS Istituto Giannina Gaslini, Genova, Italy; 3 Muscular and Neurodegenerative Disease Unit, Ospedale Pediatrico Bambino Gesù, Rome, Italy; 4 Metabolic and Neuromuscular Unit, Meyer Children Hospital-University of Florence, Florence, Italy; 5 Unit of Medical Genetics and Neurogenetics, Fondazione IRCCS Istituto Neurologico Carlo Besta, Milan, Italy; 6 Department of Pathophysiology and Transplantation, University of Milan, 20122 Milan, Italy; 7 Department of Clinical and Experimental Medicine, Neurological Institute, University of Pisa, Pisa, Italy; 8 Metabolic Unit, Ospedale Pediatrico Bambino Gesù, Rome, Italy; 9 UOC Neurofisiopatologia, Fondazione Policlinico Universitario A. Gemelli IRCCS, Rome, Italy; 10 Dipartimento Universitario di Neuroscienze, Università Cattolica del Sacro Cuore, Rome, Italia; 11 Molecular Medicine, IRCCS Fondazione Stella Maris, Pisa, Italy; 12 Pediatric Clinic IRCCS Istituto Giannina Gaslini, Genova, Italia

**Keywords:** Leigh syndrome, Mitochondrial disease, Childhood, Basal ganglia

## Abstract

**Background:**

Leigh syndrome (LS) is a progressive neurodegenerative disorder associated with primary or secondary dysfunction of mitochondrial oxidative phosphorylation and is the most common mitochondrial disease in childhood. Numerous reports on the biochemical and molecular profiles of LS have been published, but there are limited studies on genetically confirmed large series. We reviewed the clinical, imaging, biochemical and molecular data of 122 patients with a diagnosis of LS collected in the Italian Collaborative Network of Mitochondrial Diseases database.

**Results:**

Clinical picture was characterized by early onset of several neurological signs dominated by central nervous system involvement associated with both supra- and sub-tentorial grey matter at MRI in the majority of cases. Extraneurological organ involvement is less frequent in LS than expected for a mitochondrial disorder. Complex I and IV deficiencies were the most common biochemical diagnoses, mostly associated with mutations in *SURF1* or mitochondrial-DNA genes encoding complex I subunits. Our data showed *SURF1* as the genotype with the most unfavorable prognosis, differently from other cohorts reported to date.

**Conclusion:**

We report on a large genetically defined LS cohort, adding new data on phenotype-genotype correlation, prognostic factors and possible suggestions to diagnostic workup.

## Background

Leigh syndrome (LS), also known as subacute necrotizing encephalopathy [1, 2], is a progressive neurodegenerative disorder associated with primary or secondary dysfunction of mitochondrial oxidative phosphorylation [3–5]. LS is the most common mitochondrial disease in childhood [6, 7]. Clinical manifestations include psychomotor regression or retardation and signs of brainstem dysfunction, such as respiratory disturbance, nystagmus, ophthalmoplegia or dysphagia [8–10]. Symptoms often start in infancy, and many patients do not survive into childhood. LS was originally defined neuropathologically by bilateral necrotic lesions in the basal ganglia and/or brainstem [1, 11]. Lesions can now be observed in vivo with brain magnetic resonance imaging (MRI) [12–14]. LS is clinically diagnosed based on typical manifestations and neuroimaging, accompanied by elevated lactate in serum or cerebrospinal fluid (CSF). The clinical diagnosis is followed by enzyme assays on available tissues (fibroblasts or muscle) and/or genetic analysis to confirm the biochemical and molecular background [5, 15].

Several reports on the biochemical and molecular profiles of LS have been published, but there are limited studies on comprehensive evaluation and clinical genotype–phenotype correlations in broad case series [16–22]. More recently some authors included part of previously reported series in updated cohorts [23–25].

The aims of the study are to describe clinical and molecular findings of LS in a large series of Italian patients to define phenotype-genotype correlation, to identify prognostic factors and to improve diagnostic work-up.

## Materials and methods

We reviewed the data of all the 122 clinically, biochemically and/or genetically LS defined patients collected in our “Nation-wide Italian Collaborative Network of Mitochondrial Diseases” database from 2010 to 2019, diagnosed and followed up by six tertiary paediatric Centers with expertise in mitochondrial disorders.

The diagnosis was established based on the following findings: (1) clinical history suggestive for neurodegenerative disease: worsening of onset symptom or association of more than one symptom in the clinical picture; (2) focal and/ or bilateral brain MRI symmetric lesions in the deep grey matter regions, including basal ganglia, thalamus, brainstem and cerebellum; (3) biochemical diagnosis of mitochondrial respiratory chain (RC) enzyme defects and/or positive molecular analysis of genes related to LS.

The data collected in our database included: family history, age of onset, neurological and multiorgan symptoms and signs during the disease course, outcome, brain MRI patterns as reported by radiologists of each Center, metabolic profile (lactate values in plasma and CSF), biochemical analysis of RC complexes in muscle homogenate and in cultured fibroblasts and molecular data. Our study is retrospective, therefore not all data are available for all patients. The frequency of follow up evaluations was once/twice a year.

The clinical section of our web-based database includes “yes or no” dichotomic items agreed by all Centers in a preliminary consensus phase. The consensus phase was specifically designed to include the clinical features known to be relevant in mitochondrial medicine.

The database establishment and its use for scientific purposes were permitted by the local Ethical Committees of each Centers, which obtained written informed consent.

Thirty four patients of the whole cohort have been included in prior reports [26–38]

### Standard protocol approvals, registrations, and patient consents

The Institutional Ethics Committee at each center approved the study. Written informed consent was obtained from all patients or their tutors, and has been performed in accordance with the ethical standards as laid down in the 1964 Declaration of Helsinki.

## Results

A total of 122 patients with LS were enrolled in the study. Family history was reported in 117/122, age and symptoms at onset in 120/122 and 116/122 respectively, follow-up data in 108/122, plasma and CSF lactate levels in 113/122 and in 46/122, respectively.

Clinical features at the time of diagnosis and MRI pattern necessary to define “Leigh syndrome” were available in all patients. Biochemical profiles were reported in 121/122, molecular data in 110/122: not all patients presented both biochemical and molecular diagnosis but each patient presented one of the two.

### Demographics data and family history

There was a slight female preponderance in our cohort: 51 males, 71 females.

The majority of the patients were of European origin (94.2%, n = 115) including 110 Italian patients, 3 Albanians, 2 Romanians; the remaining cases were North-Africans (1.8%, n = 2) and from other unknown ethnic groups (4%, n = 5).

A positive family history was reported in 30 patients. Four sibling pairs were present in the LS cohort and 15 patients were reported having siblings with, undiagnosed neurodegenerative disorders Unspecified gait disturbance or retinopathy was referred in the mothers of 7 children with LS, who had never been examined before their children’s diagnosis; the milder maternal phenotype was suggestive for a mitochondrial trait (with different level of mtDNA heteroplasmy) or an X-linked inheritance (with skewed chromosome X inactivation).

The median age of onset was 3 months (range: from intrauterine to 6.6 years), with 95/120 patients (79%) presenting symptoms before 1 year of age, 13 between 1–2 years, 6 between 2–3 years, 5 between 3–6 years, and a single child after 6 years of age. In 3 patients prenatal signs were reported (intrauterine growth restriction in 2, oligohydramnios in 1).

**Fig. 1:**
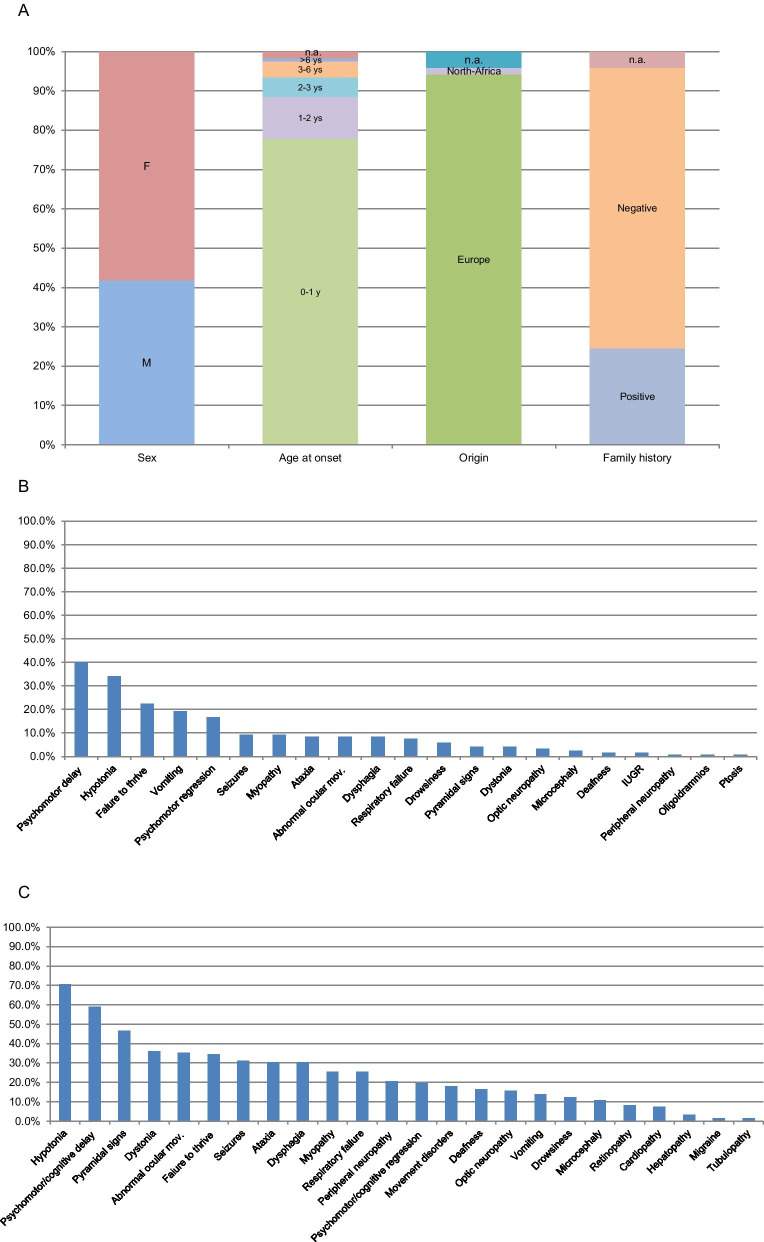
Patients' demographics and clinical findings. **A** Demographics and family history data; **B** frequency of symptoms at onset; **C** frequency of symptoms at time of diagnosis

Demographics and family history data have been summarized in Fig. 1A.

### Clinical findings at onset and throughout the disease course

The majority of patients presented more than one symptom at onset, the most common were: psychomotor delay (40%, 48/120 pts), hypotonia (34%, 41/120 pts), failure to thrive (22.5%, 27/120 pts) (Fig. 1B). In 40% of cases clinical onset was referred associated with trigger factors (e.g. infection, fever). None of the patients presented organ involvement as onset symptom.

Over the time all the patients developed a complex clinical picture characterized by the association of several symptoms. The most common neurological features were represented by hypotonia (70%) and development delay or cognitive involvement (59%), followed by pyramidal signs (46.7%), dystonia (36%), abnormal ocular movements (35.2%), failure to thrive (34.4%), seizures (31.1%), ataxia (30.3%) and dysphagia (30.3%) (Fig. 1C).

Several seizure types were observed ranging from myoclonic, tonic or spasms to severe epileptic encephalopathies with drug resistant seizures. Neuromuscular involvement was present with signs of myopathy in 25.4% and of peripheral neuropathy in 20.5%. Sensorineural deafness was reported in 16.4% of patients, optic atrophy and retinopathy in 15.6% and 8% respectively. Metabolic crisis and vomiting were observed in 13.9%. Respiratory failure due to central involvement was reported in 25.4%. A minority of patients was affected by multiorgan involvement: 9 pts (7.3%) suffered from cardiomyopathy, 4 (3.3%), from hepatopathy and 2 (1.6%) from tubulopathy.

### Lactate values

In 113 patients plasma lactate levels were measured and in 69% of them resulted to be elevated; in 46 of these 113 patients, lactate level was also analyzed in CSF and was elevated in 80.4% of them (37 patients).

Interestingly, 7 patients presented normal lactate levels both in plasma and CSF: 4 were genetically defined, harboring mutations in the *MT-ND5*, *MT-ATP6*, *BCS1L*, *GFM1* genes, and 3 patients, lacking a defined genetic diagnosis, showed one of the following biochemical defects: pyruvate dehydrogenase (PDH) deficiency, complex III deficiency and multiple complexes deficiency (I–III).

### Neuroimaging findings

Bilateral brainstem grey matter or basal ganglia lesions are included in the diagnostic criteria of LS and are therefore present in all our cases. The majority of the patients showed involvement of more than one site. Almost all had lesions in basal ganglia (110/122 pts, 90.2%); conversely, thalami or subthalami were affected in 41.8% of our cohort. At brainstem level, pontine and mesencephalic grey matter was involved in 62.3% of patients (76/122 pts) being the periaqueductal region the most commonly affected (in 23 out of 76 patients), whereas bulbar grey matter lesions were disclosed in 18.9% of our series (23/122 pts). Less frequently lesions were found in the dentate nuclei of the cerebellum (28/122 pts, 22.9%).

In addition to bilateral and profound grey matter involvement, other radiological features were reported: white matter lesions (34/122 pts, 27.9%), cortical atrophy (22/122 pts, 18%), cerebellar atrophy (14/122 patients, 11.5%) subcortical atrophy (7/122 patients, 5.7%), hypogenesis of corpus callosum (3/122 patients, 2.4%), cystic or vacuolated lesions (3/122, patients 2.4%), and cortical malformation (one patient, 0.8%).

**Fig. 2:**
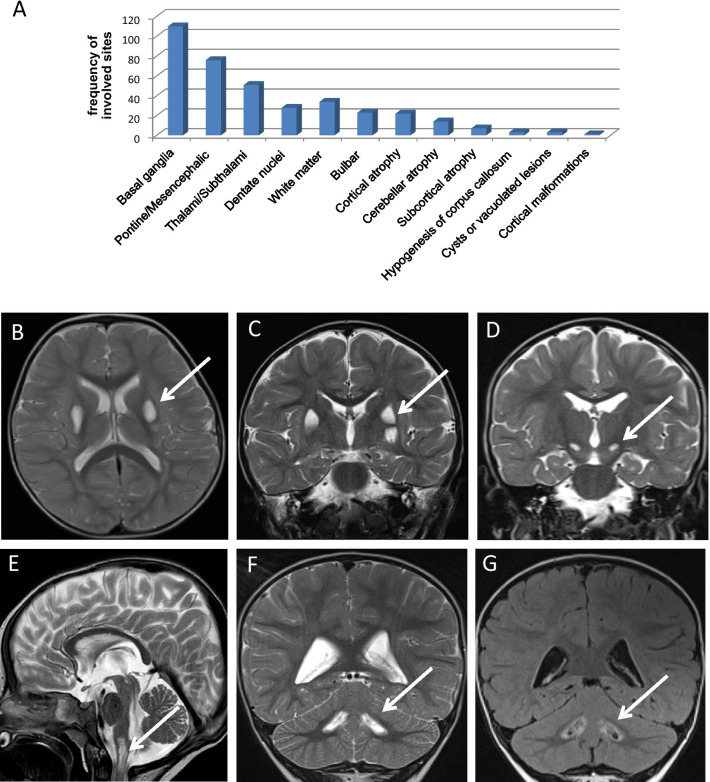
MRI findings. Graph depicts frequency of brain MRI involved regions in our LS cohort (**A**). Supratentorial pattern: axial (**B**) and coronal (**C**) T2WI sequences show hyperintensities of putamen in a patient with complex I deficiency associated to *MT-ND3*; supra and subtentorial pattern: coronal (**D**) and sagittal (**E**) T2WI sequences show hyperintensities in subthalami (**D**) and brainstem (**E**) in a patient with complex IV deficiency associated to *SURF1*; subtentorial pattern: coronal T2WI (**F**) and FLAIR (**G**) show hyperintensities (**F**) and cavited pattern (**G**) in dentate nuclei in a patient with complex I deficiency associated to *NDUFAF6*

The frequency of the abnormal brain regions at MRI and some examples are reported in Fig. 2.

### Biochemical and genetic diagnosis

**Fig. 3:**
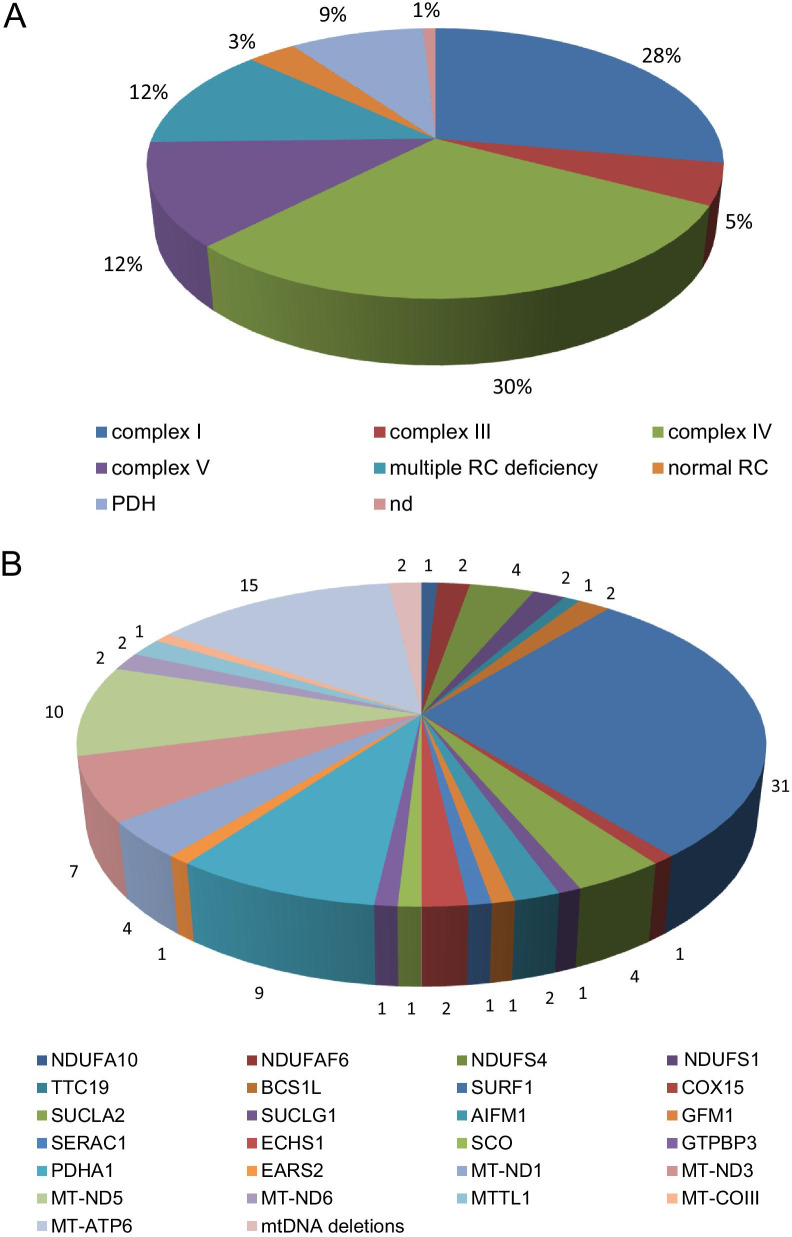
Biochemical and molecular diagnoses. Percentage of biochemical diagnoses (**A**); number of cases for each molecular diagnosis (**B**)

A biochemical assay to measure RC enzyme complex activities was performed for 121 patients in muscle homogenate or cultured fibroblasts or both (Fig. 3). The most common biochemical diagnoses were: isolated complex IV (36 patients, 29.5% of whole series) and complex I (34 pts, 27.9%) deficiencies, followed by isolated complex V deficiency (15 pts, 12.3%), multiple RC defects (15 pts, 12.3%), PDH deficiency (11 pts, 9%) and isolated complex III deficiency (6 pts, 5%). Four patients (3.3% of total) presented normal RC enzyme activity. In only one case, harboring mutations in *EARS2*, biochemical studies were not performed.

Genetic diagnosis was obtained in 110/122 patients (90.1% of whole series, Fig. 3). nuclear DNA (nDNA) mutations were more frequent than mitochondrial DNA (mtDNA) mutations (54.9% vs. 35.2%).

Molecular diagnosis was achieved in 52% of cases performing targeted gene analysis (17 mtDNA and 40 nDNA-related genes) suggested by biochemical profile, whereas in 41% using targeted next generation sequencing custom panel containing nuclear genes responsible for mitochondrial disorders or sequencing of the whole mtDNA. Whole exome sequencing was performed in 7% of cases. The most common molecular diagnoses were represented by mutations in *SURF1* and mtDNA genes encoding complex I subunits, disclosed in 28% and 23% of whole case series respectively, followed by defects in *MT-ATP6* (14%), nuclear DNA genes encoding complex I subunits (9%), and *PDHA1* (8%). In patients with complex I deficiency, defects in mtDNA were present in the majority of cases (25/34) (compared to nDNA): 10 patients with mutations in *MT-ND5*, 7 in *MT-ND3*, 4 in *MT-ND1*, 2 in *MT-ND6* and 2 with mutations in *MT-TL1*.

Details of biochemical and molecular data of our cohort are shown in Table 1, including the subdivision into 3 groups: cases due to mutations in nuclear DNA genes, cases related to mitochondrial genome, cases with biochemical diagnosis in which the genetic defect was not identified.

**Table 1:** Details of biochemical and molecular data

Biochemical diagnosis		Molecular diagnosis			
RC/PDH complexes deficiencies	n pts	Genetic defect	Inheritance	n pts	genome (tot pts)
Complex I	9	*NDUFA10*	AR	1	**nDNA (67 pts)**
		*NDUFAF6*	AR	2	
		*NDUFS4*	AR	4	
		*NDUFS1*	AR	2	
Complex III	3	*TTC19*	AR	1	
		*BCS1L*	AR	2	
Complex IV	32	*SURF1*	AR	31	
		*COX15*	AR	1	
Complexes I–III–IV	9	*SUCLA2*	AR	4	
		*SUCLG1*	AR	1	
Complexes III–IV		*AIFM1*	XL	2	
Complexes I–IV		*GFM1*	AR	1	
Complexes I–II–IV		*SERAC1*	AR	1	
Normal RC	4	*ECHS1*	AR	2	
		*SCO1*	AR	1	
		*GTPBP3*	AR	1	
PDH	9	*PDHA1*	XL	9	
n.a.	1	*EARS2*	AR	1	
Complex I	25	*MT-ND1*	Maternal	4	**mtDNA (43 pts)**
		*MT-ND3*	Maternal	7	
		*MT-ND5*	Maternal	10	
		*MT-ND6*	Maternal	2	
		*MT-TL1*	Maternal	2	
Complex IV	1	*MT-COIII*	Maternal	1	
Complex V	15	*MT-ATP6*	Maternal	15	
Complexes I–III–IV	2	mtDNA deletions	Unknonwn	2	
Complex III	3	/			**n.a. (12 pts)**
Complex IV	3	/			
Complexes I–III	4	/			
PDH	2	/			

*RC* respiratory chain, *PDH* pyruvate dehydrogenase, *nDNA* nuclear DNA, *mtDNA* mitochondrial DNA, *n.a.* not available, *AR* autosomal recessive, *XL* X-linked

Bold indicates the title of informations reported in each column

### Followup and outcome

Follow-up and outcome data were available for 108 patients, 14 cases were lost at follow up. Each patient was treated with similar mitochondrial cofactors cocktail [39].

The median follow-up time was 3.3 years (range 2 months–18 years). Thirty one (28.7%) patients remained neurologically stable. In 9/31 patients (8.3%) with the longer-term follow up of 12 months, a slight improvement of attention and “energy” was reported. Progressive neurological deterioration was reported in 68 patients (63%) and 44 of them died.

**Fig. 4:**
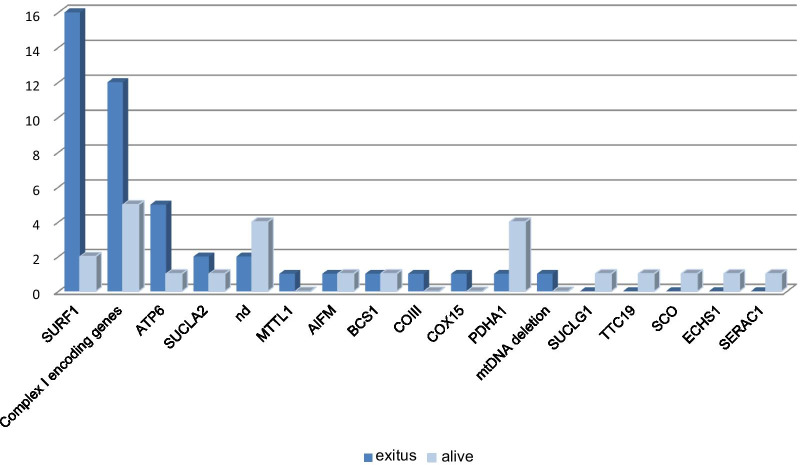
Molecular diagnosis in patients with unfavorable outcome

The majority of deceased patients presented early onset in the first year of life (42/44 pts), and complex MRI patterns with involvement of both supra and subtentorial grey matter (33/44 pts). *SURF1* resulted the most common gene associated with exitus (36% of these cases; 16/44 pts), followed by complex I-mtDNA genes and *MT-ATP6* (Fig. 4). The cause of death was respiratory failure in 19 patients, unknown in the remaining cases.

## Discussion

Our collaborative national study reports clinical and molecular data of 122 Italian patients from 6 paediatric Centers collected in the “Nation-wide Italian Collaborative Network of Mitochondrial Diseases” database.

Our study is retrospective and this could be considered a limitation, but only a few data were not available for all patients: we believe that this does not affect the main conclusions of our study.

In the last years LS case series have been reported [17–22] and some authors included part of previously reported series in updated cohorts [23–25]. We reviewed literature data (Table 2) and compared them with our results in order to evaluate possible new insights from our experience to improve knowledge on the most frequent mitochondrial disease in childhood.

**Table 2:** Biochemical and molecular data of previously published LS series

References	No of reported patients	age at onset (median age)	No of patients with available biochemical data	No of patients with molecular diagnosis	Most common biochemical profile	Most common molecular diagnosis
[17]	75	4 ys 6 ms	64	23	Complex I deficiency	*MT-ND5*
[18]	130	7 m	110	77	Complex I deficiency	*MT-ATP6*
[19]	17	n.a.	17	17	Complex V deficiency	*MT-ATP6*
[20]	39	2 ys 4 ms	32	11	Complex I deficiency	*MT-ND3*
[21]	106	9 ms	75	41	Complex I deficiency	*MT-ATP6*
[23]	96	5 ms (nDNA), 11 ms (mtDNA)	74	96	Complex I deficiency	*MT-ATP6* and *SLC19A3*
[24]	64 (of which 37 from Lee et al. 2016)	n.a.	n.a.	41	n.a.	*MT-ATP6*
[25]	166 (of which 106 from Ogawa et al. 2017)	n.a.	153	103	Normal	*MT-ATP6*
[22]	110	9 ms	92	25	Complex I deficiency	*MT-ATP6* = *MT-ND5* = *MT-ND3*
This report	122	3 ms	121	110	Complex IV deficiency	*SURF1*

Onset before 1 year of age is common in LS [18, 21–23]: in only two previously reported cohorts the median age of onset was older [17, 20]. Our data confirm the prevalent onset before 1 year of age, but the median age of 3 months is earlier than previous ones described in similar studies.

The majority of our patients presented more than one symptom at onset; the most common were psychomotor delay (40%), hypotonia (34%), and failure to thrive (22.5%). Developmental delay and hypotonia were likewise reported in others series. Conversely, failure to thrive was not observed as symptom onset and was reported only in 10.2% of cases in Sofou et al. 2014 [18]. In our series, about 1 out of 4 patients presented onset with failure to thrive. Our experience underlines that we need to consider LS in the differential diagnosis of this sign, especially if associated with apparently unspecific psychomotor delay. Interestingly, visceral involvement was never reported as an onset symptom.

During disease course, we observed that patients presented with a complicated clinical picture characterized by association of several neurological signs: pyramidal signs, dystonia, abnormal ocular movements, epilepsy and ataxia.

In spite of basal ganglia involvement, extrapyramidal signs were less common that pyramidal tetraparesis (36% of cases vs. 46.7%), according to previously reported data [20, 23]. Epilepsy was present in 31.1% of patients, similarly to other series: 25% in Ma et al. 2013 [17], 39% in Sofou et al. 2014 [18], 32% in Ogawa et al. 2017 [21], 21% in Hong et al. 2020 [22] and did not represent the major clinical problem. Epilepsy phenotype in mitochondrial diseases has also been described in well-defined case series [40–42]. Accordingly, in our cohort several seizures types were observed, ranging from focal seizures to recurrence of episodes of status epilepticus and epilepsia partialis continua, and more rarely myoclonic and tonic seizures or spasms and severe epileptic encephalopathies with drug resistant seizures.

Neuromuscular involvement has been previously described in a few cohorts and more frequently reported as unspecified “muscle weakness” [18, 20, 22]. In our cohort, neuromuscular involvement was clinically and instrumentally defined as myopathy in 25.4% or peripheral neuropathy in 20.5% of cases. Peripheral neuropathy is known as part of the clinical picture in mitochondrial disorders and has been already reported in 12.4% of the whole cohort of the Italian Network of Mitochondrial Diseases, including paediatric and adult cases [43]. Our data suggest a major frequency in childhood as compared to adulthood and underline that nerve conduction studies should be part of the early evaluation of children with undefined neurodegenerative diseases, even in absence of suspicious clinical signs.

A minority of our patients was affected by multiorgan failure. Cardiac involvement was reported in other large series ranging from 8.2 to 16% [22, 23]. In a small cohort of 14 patients, cardiomiopathy was reported in 21% [44], that decreased to 9.4% in an updated series [24]. Data on liver failure are available in few papers: 21% in a cohort of 14 cases [44] and 12.3% of another series of 130 children [18]. Renal involvement was described in a minority of patients ranging from 5.4% [18] to 7.3% [22]. According to other series, also in our population cardiomyopathy is the most common association, compared to hepatopathy and nephropathy, while global multiorgan involvement in our cohort was very rare and less frequent if compared to other series. Interestingly, the analysis of our population and the review of previously reported paediatric mitochondrial diseases large series[45–47], disclosed multiorgan involvement in no more than 16% of patients, suggesting that in childhood primary organ involvement is less frequent than expected.

Lactate levels were elevated in 69% (plasma) and in 80.4% (CSF) of patients. In 15.1% (7 ts), lactate levels were normal in both specimens: these cases included 4 children with genetically confirmed diagnosis and 3 with biochemical diagnosis and undefined molecular defect. In other published series, abnormal lactate levels in plasma or CSF were reported as a frequent but not constant laboratory finding; nevertheless, correlations with biochemical defects or genetic confirmed diagnosis were not reported. Our data confirm that high lactate levels are not a constant laboratory finding in genetically confirmed LS and that additional more specific biomarkers are required.

Only few papers described in detail MRI data on large populations. Sofou et al. 2018 [23] reported correlations between MRI pattern and nDNA or mtDNA gene defects in 96 patients, and concluded that no significant differences were seen between these two major groups; isolated infratentorial involvement was clarified in NDUF-associated cases and was less common (3/12). Hong et al. 2020 [22] analyzed MRI findings in 110 patients. Frequency of basal ganglia involvement was similar to our cohort: 94.5% versus 90.2% in our study. Thalamic alterations were more frequent in our population (41.8% vs. 34.5%), whereas we observed bulbar and white matter signal abnormalities less frequently (18.9% vs. 27.3% and 27.9% vs. 38.2%, respectively). We reported pontine and/or mesencephalic involvement in 62.3%; this data is not comparable because Hong et al. divided alteration in midbrain (40.0%) and pons (24.5%). Regarding the cerebellum, we distinguished dentate nuclei alterations (22.9%) and atrophy (11.5%), while Hong et al. reported unspecified cerebellar involvement in 37.3%. Anyway, cerebellum involvement was less common in our cohort. The same is for evolution of cerebral atrophy observed in 58.2% of patients described by Hong et al., whereas we distinguished cortical atrophy in 18% and subcortical atrophy in 5.7%.

In order to analyze MRI findings correlations with the biochemical and molecular diagnoses, we identified 3 principal MRI patterns: supratentorial (basal ganglia, thalami, subthalami), subtentorial (brain stem, dentati nuclei) and supra-plus subtentorial.

Supra-plus subtentorial pattern was the most common in cases related to both nDNA and mtDNA (67% and 74% respectively), isolated supratentorial was observed in 29% of nDNA and 21% of mtDNA related cases. Subtentorial pattern was rare in both groups: 4% of nDNA cases and 5% of mtDNA cases.

No other differences were evident looking at the biochemical diagnosis and specific gene involved, and no correlation between MRI pattern and genotype was clearly evident. Nevertheless, for several genes, the number of LS patients is very limited and this probably my hamper the identification of specific genotype–phenotype correlations.

In our series biochemical profile was assessed in 121/122 with RC defects present in 117. The most common biochemical diagnosis was isolated complex IV (29.5%) followed by complex I (27.9%) deficiency.

In previous series, isolated complex I deficiency has been already reported as the main biochemical diagnosis in LS [17, 18, 20–23], conversely isolated complex IV was reported only in a few reports [22, 25]: our data disclosed complex IV deficiency as the most common biochemical diagnosis in a LS cohort for the first time todate.

Moreover, we identified PDH deficiency in 9%; this finding has been previously reported only in the cohort described by Sofou et al. 2018, in 6% of cases. Even if PDH deficiency is known as a cause of LS, it was seldom reported before in LS cohorts, probably because the corresponding biochemical investigation is rarely performed. Therefore, the frequency of PDH deficiency in this disease has not been well defined, but our study suggests it may corresponds to about 5–10% of LS cases. In our series we also disclosed cases with isolated complex V (12.3%) and isolated complex III (5%), that were not reported in large cohorts, but only described by Ma et al. 2013 [17].

Finally, none of the patients presented complex II deficiency and normal biochemical profile was extremely rare in our cohort (3.3%). The latter finding could be due to specific defects affecting mitochondrial pathways not strictly linked to RC or PDH (e.g. ECSH1 mutations), as previously reported [9]. Notably, clinical presentations overlapping LS or with Leigh-like features have been described associated with mutations in nuclear genes encoding proteins with non mitochondrial localization (e.g. RANBP2, SLC39A8), and suggested to cause a secondary impairment in mitochondria without any clear RC deficiency [48].

Another possible explanation to explain the absence of RC/PDH impairment is linked to the tissue-specificity of the biochemical deficit, which is a common finding in diverse mitochondrial disorders; in predominantly neurological diseases like LS, it may hamper the identification of the biochemical defect in non-affected cells/tissues such as fibroblasts or even muscle.

Our study disclosed isolated complex IV as one of most common biochemical diagnosis in LS, it added the complex III and V defects among the biochemical diagnosis in LS, previously reported only in a single series, and identified and defined PDH deficiency in a substantial percentage of LS.

In our population, molecular diagnosis was achieved in 110/122 patients (90.1% of whole series); the most common were mutations in *SURF1* (28%) and mtDNA genes encoding complex I subunits ( 23%), followed by *MT-ATP6* (14%), nuclear DNA genes encoding complex I subunits (9%) and *PDHA1* (8%).

MtDNA genes encoding complex I subunits and *MT-ATP6* were the most common causes related to mtDNA in other series too [23–25]. Conversely, *SURF1* was rarely reported in other cohorts (7/103 in Ogawa et al. 2020, 2/41 in Lee et al. 2020). In these series, the most common nDNA genes identified were not related to OXPHOS subunit or assembly factors, and included *SLC19A3, SUCLA2* [23], *ECHS1* [25] and *IARS2* [24]. This discrepancy is possibly linked to a different prevalence of nDNA variants in these genes in distinct populations. Our experience in the diagnostic work up of clinical hypothesis for LS—suggested by early onset of several neurological signs associated with bilateral and profound grey matter regions at MRI- confirms the usefulness of biochemical assay in order to address targeted molecular analysis in specific cases.

Indeed, it suggests to prioritize the analysis of *SURF1* (for complex IV deficiency), the screening of mitochondrial genes prior to nuclear genes encoding complex I subunits (for complex I deficiency), and the analysis of *MT-ATP6* mutation (for complex V deficiency); because these are the most common genetic defects, their investigation is suggested also for cases without available biochemical data. In other isolated or combined RC enzyme defects, sequencing of the whole mtDNA and of nuclear genes associated with LS is recommended. The final step in unsolved cases is whole exome sequencing (or whole genome sequencing).

Follow up and outcome data were available in almost all patients (88% of whole cohort), and the mean follow-up time was 3.3 years. More than half of cases (63%) worsened during the course of disease history and the majority of them died in the timeframe of this study. The survival outcome seemed not influenced by the pattern of inheritance. We observed that early onset (within the first year of life), complex MRI pattern with involvement of both supra and subtentorial grey matter and *SURF1* mutations can be considered the most unfavorable prognostic factors According to previous data, the early onset [18, 22, 25] and association with brainstem lesions on neuroimaging associated with poorer survival [18], our report adds further strong evidence for this observation.

Conversely, LS with *SURF1* mutation has been reported to be associated with longer survival than other types of LS [13, 25, 49], whereas our study showed a strong genotype–phenotype correlation between *SURF1* and high risk for exitus that has not been previously reported.

## Conclusion

Our study confirmed that LS clinical picture is characterized by early onset of several neurological signs dominated by central nervous system involvement of supratentorial (with or without subtentorial) grey matter at the MRI in the majority of cases. In spite of mitochondrial diseases being typically considered as multisystem disorders, our study suggests that for LS multiorgan involvement is less frequent than expected.

Elevated lactate levels are not constant laboratory findings. Our results disclosed that isolated RC complex IV and, less frequently, complex I deficiency are the most common biochemical signature in LS, and defined PDH deficiency in a significant percentage of LS cases.

We report *SURF1*—for the first time todate- and mtDNA genes encoding complex I subunits as the most common LS genetic defects, and show *SURF1*-related LS as the genetic subtype having the most unfavorable prognosis, differently from other series reported so far.

## Data Availability

Data that support the findings of this study are available from the corresponding author (A.A.) and will be shared anonymously by request from any qualified investigator.
